# Recruitment Strategies to Engage Newcomer Mothers of African Descent in Maternal Mental Health Research in Canada

**DOI:** 10.1177/10436596221090268

**Published:** 2022-04-25

**Authors:** Deborah Baiden, Marilyn Evans

**Affiliations:** 1 University of Toronto, Ontario, Canada; 2 Western University, London, Ontario, Canada

**Keywords:** newcomer, recruitment, cultural competency in research, African, maternal mental health

## Abstract

**Introduction:**

Newcomer mothers of African descent are at risk for maternal mental stress because of inadequate social support, newcomer status, and stress of motherhood. Limited participation of newcomer African mothers in mental health research contributes to a knowledge gap in this area further impacting culturally competent health services. This article reports recruitment strategies to better engage African newcomer women in maternal mental health research.

**Methods:**

In-depth discussion of recruitment strategies, used in a qualitative descriptive study conducted with Black African newcomer mothers in Canada.

**Results:**

Ten African newcomer mothers were successfully recruited using recruitment strategies such as engagement with religious organizations, snowballing, and the use of social media.

**Discussion:**

Cultural beliefs on motherhood, resilience, and mental illness may account for hesitancy to engage in maternal mental health research. Recruitment strategies could help overcome the challenges and potentially diversify maternal mental health research in Canada through the engagement of African newcomer mothers.

## Introduction

Approximately 23% of new mothers in Canada experience symptoms of mental unwellness after birth (Statistics Canada, 2019). Several factors have shown to contribute to the experience of postpartum mental stress among new mothers including disappointment about baby’s gender, discrepancies between perceptions of pregnancy events and reality, gender-based violence, and inadequate exercise (Ghaedrahmati et al., 2017). New mothers, who are recent immigrants, face the stress of migration and settlement in a new country along with the challenges of navigating cultural differences of motherhood, diet, and socialization, resettlement in a new country, and lack of social support (Ghaedrahmati et al., 2017; Herrero-Arias et al., 2020). Visible minorities are often viewed as one monolithic group, ignoring the diversity in their ethnicities, beliefs, health practices, and health outcomes (Omenka et al., 2020). For African immigrant women, in particular, gendered and cultural aspects about womanhood and being a mother, spirituality, socioeconomic factors, and the stigma attached to mental illness impact their conceptualization of maternal mental illness in addition to the barriers they face in navigating a complex health system to access and utilize available maternal mental health and support (Babatunde & Moreno-Leguizamon, 2012; Baiden & Evans, 2021; Gardner et al., 2014). The paucity of knowledge on the maternal mental health of newcomer mothers of African descent in Canada creates challenges for health professionals. Some of these challenges include difficulty in the provision of culturally safe mental health services structured using antiracist frameworks that are tailored to the needs of African newcomer mothers. The provision of culturally safe mental health services structured using antiracist frameworks foster transformative action in the health care system. Transformative action in the health care system is health care services that are equitable, culturally appropriate, antiracist, and recognize the multidimensionality of individuals.

Research that includes societal and ethnic diversity is essential to gain a full understanding of individuals’ experience of mental health issues and to inform health services and mental health programs (Williams et al., 2013). Canada’s visible minority population is increasing rapidly, yet despite the demographic significance of this population, there is a surprising dearth of nationally representative health data on visible minorities (Khan et al., 2015). Such research is important to reveal inherent cultural nuances in the meaning and interpretation of mental illness and the preferred ways of managing it by diverse populations (Babatunde & Moreno-Leguizamon, 2012). There is a compelling need to recruit participants who are under-represented in mental health research to gain their perspective. A study by Gardner et al. (2014) reveals African immigrants in the United Kingdom have a misconception about the absence of maternal mental health issues in their home countries and note an inadequate awareness about postpartum depression. For African women living in an African country, factors impacting maternal mental health care include inadequate finances and involvement of health care workers at the community level (Nakku et al., 2016). Furthermore, African women being mothers and maintaining postpartum mental wellness in non-African context can be an isolating experience, without substantial support and with loss of culture, connection, and identity (Gardner et al., 2014).

Mental illness stigma is a significant factor associated with the underutilization of mental health services among populations of African descent (Harris et al., 2020). Inadequate knowledge, stereotyping, and prejudice against people facing mental health issues could prevent the utilization of mental health services (Corrigan et al., 2014). According to Brown et al. (2014), visible minorities are unlikely to engage in mental health research primarily due to their nonuse of mental health services and because recruitment for mental health research primarily occurs in health care facilities and/or is focused on current users of mental health services. The stigma attached to mental illness among visible minority populations who partake in mental health research extends to their families (Brown et al., 2014). Stigmatization of mental illness is influenced by cultural approval of discriminatory behaviors and experiences of prejudice from one’s social circle and toward the family of a person dealing with mental illness (Corrigan et al., 2014). As mental illness is stigmatized at the individual, family, and system levels (Corrigan et al., 2014), participating in maternal mental health research might lead to further stigmatization of the women by her family and stigmatization of her family by community.

Fête et al. (2019) suggest immigrants, being a vulnerable and hard-to-reach group, are under-represented in research. Inadequate representation of ethnic and visible minorities in health research (Redwood & Gill, 2013) impacts generalizability of research results, health equity, and ethnic specific health care services (George et al., 2014). Visible minorities have a distrust of the health care system and professionals, therefore are reluctant to seek help or support for well-being (Keefe et al., 2021; Said et al., 2021). Creating a safe space by ensuring privacy and building trust through cultural competency and antiracist care could improve access to and utilization of mental health services among Black women (Keefe et al., 2021; Said et al., 2021). Such measures could impact their representation in health research. Increasing the recruitment of visible minorities in mental health research requires identification of barriers, such as lack of trust, as well as investment of time and resources to implement effective methods for improving their acceptance of and participation in such research (Waheed et al., 2015).

The purpose of this paper is to provide an in-depth discussion of recruitment strategies for engaging African newcomer women in maternal mental health research in Canada. First, an overview of African newcomer mothers’ hesitancy to participate in maternal mental health research is presented. As well, a discussion on how mental illness stigma, intersectionality, and the resilience of African newcomer mothers influence their participation is discussed. Finally, recommendations for recruitment strategies to encourage and engage newcomer mothers of African descent in maternal mental health research are outlined. The exposition from this paper seeks to inform maternal mental health researchers on engaging newcomer mothers of African descent in Canada in future research activities.

## Method

The discussion stems from results of a qualitative descriptive study to explore the sociocultural determinants of the perception of mental health and mental health services utilization among 10 Black African newcomer women in Canada who had given birth within the past year (Baiden & Evans, 2021). Newcomer mothers in this study were described as those who have birthed a baby within the past year. Using feminist ethnography (Davis & Craven, 2016; Im, 2013; Schrock, 2013), in-depth ethnographic interviews each lasting an hour were conducted to center the voices of the African newcomer women over the course of 5 months. This study focused on the cognitive aspects of culture such as perception, which cannot be observed through participant observation. Ethnographers confirm that in-depth ethnographic interviews can adequately be used in studies focused on the cognitive aspects of culture (Forsey, 2010; Hall et al., 2012; Hockey & Forsey, 2012). The women downplayed the manifestations of maternal mental un-wellness, favored spirituality, and valued spousal and social support to embrace their new mother role. Their beliefs about motherhood symbolized a badge of strength and resilience, hence mental stress in the perinatal period was minimized, and echoed by participants as a barrier to mental health service utilization and potential maternal mental health participation.

Resilience, among African mothers in Rwanda, promotes maternal mental well-being and is fostered by the community because of the collectivist culture (Khanlou & Pilkington, 2015). Thus, the African saying that “it takes a village to raise a child” is in recognition of the fact that parenting alone could be mentally stressful and captures the value of communal social support in promoting resilience in motherhood. Health care services reflect the cultural environment (Ibeneme et al., 2017); hence, for African women who have recently migrated to a Western country, the change in culture means resilience is, primarily emphasized at the individual level. Baiden and Evans (2021) also unveiled the influence of strong Black woman (SBW) schema on Black African mothers’ willingness to participate in their research study. For example, several women who were invited expressed that mothers of African descent do not face mental stress following childbirth, and others questioned the rationale for the study. Set in Southern Ontario, our study comprised of newcomer Black women from mostly Sub-Sahara African countries who have migrated voluntarily to Canada. Initial recruitment strategies involved the traditional use of recruitment posters; however, this strategy alone was not successful due to several challenges including the need for trust building and nuances attached to language describing mental health. Restructuring recruitment strategies such as the engagement of religious organizations, leaders, and members of African immigrant groups, and using the networks of potential participants were helpful. These community-based recruitment strategies are essential due to the importance of community support to African communities. Eventually, 10 women volunteered to participate in the study who were within 1 to 12 months postpartum, and nine were married. In relation to employment, one was a full-time graduate student, two were unemployed, three were on maternity leave, and four were still working either full-time or part-time. It is important to note that these women were not employed in high-income jobs. Newcomer women to Canada are more likely than their male counterparts and Canadian-born to be low-income earners (Crossman, 2013).

There is an urgent demand for culturally competent health care professionals and increased public awareness of maternal mental health throughout the perinatal period among this group (Baiden, 2019; Baiden & Evans, 2021). The recruitment of women of African descent in clinical research is needed but remains challenging (Smith et al., 2007), leading to a lack of evidence to inform mental health services specific for this population.

## Results

### Engaging African Newcomer Mothers in Maternal Mental Health Research

African immigrant women are less likely to utilize mental health services due to their cultural beliefs that mental stress during motherhood is a sign of weakness (Babatunde & Moreno-Leguizamon, 2012). In Canada, Etowa et al. (2017) identify that the SBW schema, previously studied among African American women, could explain the health behaviors of African Nova Scotian women around utilization of health and social services to meet health needs and for support. The SBW schema is defined as a culturally distinct concept that is rooted in gendered racism and posits that, women of African descent may be more likely to rely on self, less likely to express emotional weakness, and less likely to seek help for emotional challenges than non-African women (Watson-Singleton, 2017).

There are several challenges to successful recruitment and participation of Black African women in conducting mental health research. Findings of systematic literature review (Brown et al., 2014) revealed challenges to recruitment of ethnic minority women include the worry of being labeled as mentally unwell, researchers lacking cultural expertise, participants’ suspicions of the research and time constraints. Immigrants in Canada are at risk for health inequities because of experiences of social disparities, financial difficulties, migrant experience, and discrimination (Waldron, 2010). Furthermore, participants in our qualitative study, revealed the vulnerability of a newcomer status added to their anxiety of taking part in research where they would talk about their perception of the mental health services in the new country that they are already trying to navigate. The culturally driven perception of motherhood being associated with nonstressful and positive feelings may contribute to misinterpretation or minimizing of maternal mental stress, leading to under-utilization of mental health services during the maternal period (Mamisachvili et al., 2013). These beliefs may also contribute to women’s hesitation to participate in mental health research.

Revealing barriers and facilitators of mental health and service utilization by African newcomer women are important to improve interactions with African newcomer mothers in maternal mental health research. Stigma and cultural beliefs on mental illness during the maternal period are barriers to mental health utilization among the women while, awareness, partner support, spirituality, health education, and anonymized services act as facilitators (Baiden & Evans, 2021). Understanding these facilitators could improve engagement with African newcomer women and addressing recruitment challenges in maternal mental health research.

According to Waheed et al. (2015), cultural variations among ethnic minorities require mental health research to incorporate culturally appropriate methods and modifications to produce relevant and useful evidence. Strategies to enhance recruitment and engagement of Black newcomer women of African descent in Canada for mental health research need to be tailored made and specific to their values, beliefs, and situation. Ogilvie et al. (2008) assert researchers engaging recent immigrants in health research should consider cultural differences, positionality in relation to research, and the context of recent immigrants trying to navigate a new country and health system. For our research study, we remained sensitive to and reflected on how our research positionality might impact our recruitment and engagement of potential participants for maternal mental health research. The first author acknowledged her insider position as a Black African newcomer woman in Canada and a recent new mother at the time of the study. As a recent new mother without family relatives close by, the first author faced challenges in accessing social support and relied on church members who had experience of motherhood. The insider role helped inform recruitment strategies, for instance, the use of churches and other religious organizations. However, it was important to note that the potential participants migrated from varying geographical locations from the African continent, belong to diverse tribal groups, and have different socioeconomic statuses, making the first author an outsider to some extent as well. According to Ogilvie et al. (2008, p. 67), “one is never completely an insider or an outsider in the research process.” The second author, who as a Caucasian nonimmigrant woman, may not entirely be an outsider and share similar nurturing and caregiving perspectives as a mother with participants. Furthermore, experience as health care professionals and researchers in maternal health informed the outsider role for both authors. Reflexivity was maintained by recording notes on biases and ideas and reflect on social positions of race, socioeconomic status, ethnicity, and the verbal and nonverbal reactions in a reflective journal throughout the research process (Olukotun et al., 2021).

In the recruitment and engagement of Black African newcomer women in maternal mental health research, researchers need to draw on the commonalities they share as well as acknowledge and be thoughtful of their diverse backgrounds/experiences and worldviews to create a respectful and culturally safe space for trust building and meaningful research. Redwood and Gill (2013) advocate for a nonjudgmental discourse when engaging populations underrepresented in health research. Trust-building is imperative when engaging ethnic minority populations in research to encourage participation because of their wariness of research and health care providers (Sankaré et al., 2015). African immigrant women may not disclose emotional problems to health care professionals and members of their community because of stigma and privacy concerns regarding personal issues. To research sensitive topics, such as maternal mental health with its associated stigma, trust building is imperative to creating a safe space. To mitigate unequal power balances between the researchers and participants, participants selected the venue and the mode of conducting interviews, chose their pseudonyms, and were encouraged to be involved in confirming findings as co-constructors of knowledge.

### Strategies for Recruiting Newcomers of African Descent in Maternal Mental Health Research

Several strategies were used to invite women to participate in the abovementioned qualitative study. Posters with details about the study and contact information for interested women were displayed on the bulletin boards of churches of primarily congregants of African descent. Similarly, Cudjoe et al. (2019) engaged African immigrant churches for recruitment of participants in their study on the cervical screening habits of African immigrant women in the United States. Gatekeepers in research include individuals, stakeholders, and group representatives who link researchers to eligible participants based on their understanding, belonging to the community, and trust by the community members (Andoh-Arthur, 2019). Churches in the African community act as gatekeepers as they provide access to members of this community as well as serve as valuable resources for recruitment. When recruiting participants from churches, it is necessary to first build a trusting open relationship with the church management through initial visits prior to the commencement of recruiting potential participants and collecting data (Cudjoe et al., 2019).

The first author attends a Black church and was a student member of an organization that offers support and provides access to resources appropriate for African immigrants in the local community. As a member of an African immigrant organization, she consulted the executives of the organization as part of the recruitment strategy. She built rapport by sending e-mails, making phone calls and/or scheduling in person meetings with potential gatekeepers in churches who also spoke with other gatekeepers in various cities in Southern Ontario (Baiden, 2019).

Posters advertising the study were also displayed in hair salons offering hair services to Black clientele. The digital poster was circulated on WhatsApp group pages whose membership included recent immigrant women. Administrators of social media pages were contacted for their consent to share the posters on their social media group pages. Similarly, Twamley et al. (2009) contacted media organizations to share information when recruiting for their study on maternity care among UK-born ethnic minority women. A digital version of the poster was also given on the social media platforms of the women groups in some churches with a predominantly African membership in Southern Ontario.

Snowballing was another effective recruitment strategy, where participants engaged with other newcomer women in their social network by informing them about the study and advertising the research to gain community support. The researchers verbally communicated details on the study to these potential participants if contacted. Many of the women were recruited through casual conversations about the study among newcomer mothers. The use of casual conversations as a recruitment and engagement strategy when conducting sensitive research with populations of African descent has been successful in the United States (Cudjoe et al., 2019; Sankaré et al., 2015). In the United Kingdom, majority of ethnic minority women recruited for a maternity care study were contacted using casual conversations among personal contacts of participants (Twamley et al., 2009). Maternal mental health researchers can adapt this strategy to encourage participation from African newcomer mothers. Having recently migrated to Canada, African newcomer women will seek and form social networks for support through family, friends, and community networks (Hynie et al., 2011). Furthermore, African newcomer women often live in neighborhoods with immigrant populations, may be part of religious associations, and may have family and/or friends who have lived in the host country longer than they have. Sankaré et al. (2015) recruited majority of participants using snowballing and casual conversations with people in their circle, as this strategy encourages acceptance and participation.

Unexpected challenges occurred during the initial stages of recruitment women to our study. Cultural nuances in language and negative interpretation and stigma linked to the word “mental health/illness” created a barrier to recruitment. We amended the language used in advertising the study and in our conversations with Black African newcomer women. We substituted “mental illness” with “mental stress, stress, and emotional stress.” In addition, emotional well-being and feelings after childbirth were used, as the women did not equate mental illness as something new mothers would experience. This amendment improved participation in the study and confirms findings from Brown et al. (2014) that populations of ethnic and visible minorities may be unwilling to participate in mental health research because of the fear of mental illness stigma from their social network. According to Davis and Craven (2016) attention to discourse, language, and associated interpretations by potential participants regarding sensitive topics is vital when conducting feminist ethnography. The cultural nuances in language and their impact on willingness of this population to partake in research should be considered.

## Discussion

### The Implications of Intersectionality, Mental Strength, and Stigma on Maternal Mental Health Research

Our study suggests that intersectionality of gender, race/ethnicity, culture, stigmatization of mental illness, and mental strength contributed to the Black African newcomer mother’s hesitancy to participate in maternal mental health research. Intersectionality, first devised by Kimberle Crenshaw, originates from Black feminism and acknowledges the impact of multiple social identities on health inequities (Bowleg, 2012). For instance, based on research in the United Kingdom, Babatunde and Moreno-Leguizamon (2012) advocate that health care providers consider the cultural context, family dynamics, and newcomer status of African newcomer women. In addition, immigration status may influence newcomers’ hesitancy in the sharing of personal information due to the lack of trust and fear of authorities using this information to harm them (Ogilvie et al., 2008). The intersection of gender, race/ethnicity, and culture shape social position and decision-making around the use of mental health services for individuals of African descent and may also influence Black African newcomer women’s decision to participate in mental health research and access health services; a health service under-utilized in populations of African descent (Harris et al., 2020). Although the participants in our study (Baiden, 2019; Baiden & Evans, 2021) had common social identities, including African ethnicity, Black racial identity, recent immigrants in a western country, being new mothers, and identifying as women, their experiences, from these multiple social identities, were unique. Viruell-Fuentes et al. (2012) emphasize health researchers need to use intersectionality to guide the understanding of the concurrent interactions of immigrants’ multiple social identities and how they impact health outcomes and experience of health inequities. This insight is valuable to create effective and targeted recruitment strategies for including under-researched and visible minorities populations in health research.

The SBW schema is a phenomenon to describe Black women’s reliance on mental strength and their censoring of symptoms of mental illness to satisfy cultural and societal beliefs of motherhood and overcoming stress, thus affecting their willingness to use maternal mental health services (Abrams et al., 2019; Etowa et al., 2017). The SBW schema provides an understanding of African newcomer women’s resilience and mental strength in maintaining postpartum mental wellness (Baiden, 2019; Baiden & Evans, 2021). In addition, Abrams et al. (2019) posit that the joint contribution of sociocultural factors and past events surrounding gendered racism results in the SBW schema. In conducting maternal mental health research, researchers need to unpack how cultural beliefs on mental health, societal, and cultural expectations of motherhood, the SBW (Strong Black Woman) schema, a precarious immigration status, and work and family responsibilities intersect to impact on African newcomer women’s willingness to participate.

Immigrant mothers of African descent in the United Kingdom believed maternal mental stress can be treated by spirituality, social support, and bonding with baby, and downplayed symptoms of postpartum mental unwellness because of associated stigma (Gardner et al., 2014). The women also reported on the nonexistence of mental unwellness after childbirth in their home countries in Africa (Gardner et al., 2014). African mothers living in Uganda face barriers to knowledge about the recognition and treatment of maternal mental health and use spirituality to explain the causes of maternal mental health (Nakku et al., 2016). In our study, the notion that mental unwellness was not present when embracing motherhood affected the recruitment and participation of eligible participants. One strategy to mitigate this, as described earlier, is the amendment of the language used in informing potential participants about the study and of their eligibility.

Intersectionality and health-related stigma affect populations uniquely based on their marginalized social identities (Rai et al., 2020). According to Rai et al. (2020), intersectional difficulties associated to gender and socioeconomic status were more prone to health-related stigma. For our study, intersectional hurdles related to the resilience of being a SBW and having a newcomer status could precipitate maternal mental health stigma. In addition, a precarious immigration status of an immigrant might contribute to reservations about participating in research (Fête et al., 2019). This could translate into African newcomer women’s hesitancy to participate in maternal mental health research because of their intersectional experiences. Caiola et al. (2014) mention that motherhood is considered a social construct shaped from a historical and cultural background, without a universal definition, and rooted in the intersectional background of the mother. Motherhood, for African newcomer women, is perceived to be a joyous period devoid of maternal mental stress. Thus, maternal mental health research may not seem culturally relatable for an African newcomer woman, hence the hesitancy to participate.

## Conclusion

There are several hurdles to the recruitment and engagement of newcomer mothers of African descent in maternal mental health research. Influential factors such as the intersection of gender, race/ethnicity and culture, resilience, and mental health stigma may add to the hesitancy of African newcomer women to participate in maternal mental health research. Recruitment strategies such as engaging Black churches and hair salons, using snowballing and informal conversations, and the considerations of the cultural nuances of language are effective in improving research participation by this population. An increase in participation of African newcomer women in maternal mental health research would lead to more representation and the diversification of literature. Such research would in turn translate to informing equitable health policies and programs, and the provision of culturally safe health care.
